# Measuring Near Plasma Membrane and Global Intracellular Calcium Dynamics in Astrocytes

**DOI:** 10.3791/1142

**Published:** 2009-04-26

**Authors:** Eiji Shigetomi, Baljit S. Khakh

**Affiliations:** Departments of Physiology and Neurobiology, David Geffen School of Medicine, University of California, Los Angeles

## Abstract

The brain contains glial cells.  Astrocytes, a type of glial cell, have long been known to provide a passive supportive role to neurons. However, increasing evidence suggests that astrocytes may also actively participate in brain function through functional interactions with neurons.  However, many fundamental aspects of astrocyte biology remain controversial, unclear and/or experimentally unexplored. One important issue is the dynamics of intracellular calcium transients in astrocytes. This is relevant because calcium is well established as an important second messenger and because it has been proposed that astrocyte calcium elevations can trigger the release of transmitters from astrocytes. However, there has not been any detailed or satisfying description of near plasma membrane calcium signaling in astrocytes. Total internal reflection fluorescence (TIRF) microscopy is a powerful tool to analyze physiologically relevant signaling events within about 100 nm of the plasma membrane of live cells. Here, we use TIRF microscopy and describe how to monitor near plasma membrane and global intracellular calcium dynamics almost simultaneously. The further refinement and systematic application of this approach has the potential to inform about the precise details of astrocyte calcium signaling. A detailed understanding of astrocyte calcium dynamics may provide a basis to understand if, how, when and why astrocytes and neurons undergo calcium-dependent functional interactions.

**Figure:**
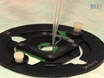


## Protocol

### EXPERIMENTAL PROCEDURES

The experimental procedure consists of two key parts that are described in a step wise manner below.

### Part 1: PREPARING HIPPOCAMPAL ASTROCYTE CULTURES

Briefly, mixed hippocampal astrocyte-neuron cultures were prepared using a well established protocol^1,2,3^. We optimized the procedure to yield healthy cultured astrocytes. All the procedures listed below should be carried out in a sterile environment such as a laminar flow hood.

**Preparing coverslips**

- 22 mm coverslips (VWR, 48380-068)
- Poly-D-Lysine (PDL, Sigma P0899), aliquots (1 mg/ml)
- Laminin aliquots (20 μg/ml): add 49 ml of sterile water to 1 mg/ml laminin solution (Sigma L2020), make 1.2 ml aliquots and store at -20°C

1. Dissolve PDL in sterile water (50 μg/ml)
2. Autoclave the coverslips, rinse with sterile water and place in PDL (20 ml for 100 coverslips). Leave at room temperature overnight.
3. Replace PDL with sterile water (3 extensive washes). Then dry the coverslips and store at 4°C.
4. The day before plating the astrocytes, place individual coverslips in a sterile well on a 6-well plate (Multiwell Flat-Bottom Plates with Lids, Sterile from BD Bioscience). Put 400 μl of laminin on each coverslip and incubate overnight, in the incubator to avoid evaporation.

 

**Dissection**

*Dissection media:*

- 500 ml Earle’s Balanced Salt Solution (EBSS) (1X), liquid (Invitrogen, 14155-063)
- 5 ml HEPES solution (Sigma, H0887)

 

*Hippocampal media:*

- 412.5 ml Minimum Essential Medium (MEM) (1X), liquid Contains Earle's salts, but no L-glutamine or phenol red (Invitrogen, 51200-038)
- 10 ml glucose (Sigma, D-(+)-GLUCOSE ANHYDROUS SIGMA ULTRA G7528) 1 M in MEM
- 5 ml Penicillin-Streptomycin (Invitrogen, 15140-122)
- 5 ml Sodium pyruvate solution (Sigma, S8636)
- 12.5 ml HEPES solution 1 M (Sigma, H0887)
- 5 ml N-2 Supplement (100X), liquid (Invitrogen, 17502-048)
- 50 ml Horse Serum, Heat-Inactivated (Invitrogen, 26050-088)

 

 

*Papain solution:*

- Add 5 ml of dissection media into Worthington PAPAIN-022 vial (LK003178; final concentration, 20 U/ml) and incubate  at 37°C for 60 min.

 

1. Decapitate rat pups aged P1 (typically 2 pups) following guidelines vetted and approved by national regulations and your local Institutional Animal Care and Use Committee.
2. Remove skin and skull and place brain in Petri dish filled with cold dissection media.
3. Removed meninges, dissect both hemispheres and dissect hippocampi.
4. Chop the hippocampi into ~1X1 mm pieces and digest in papain solution at 37°C for 11-13 min.
5. Once the pieces of tissue have settled, remove papain carefully and add 5 ml of hippocampal medium to remove all traces of the enzyme. Repeat this step and resuspend in hippocampal medium (2 ml).
6. Triturate the cells ~ 5 times until there are no clumps left, with three flame-polished pipettes of progressively smaller bores (1000 μm, ~500 μm and ~300 μm).
7. Once triturated, pass the cells through a cell strainer (pore size, 70 μm, BD Bioscience). Count the cells in 10 μl of suspension. Adjust the volume of hippocampal medium in order to have 400000 cells/ml.
8. Aspirate the laminin off the coverslips, and before the covers can dry, plate 200 μl of the cell suspension per coverslip.
9. Leave them to attach for 60 min in the incubator, and then add 2 ml of hippocampal medium per well.
10. The next day, aspirate old hippocampal medium to remove dead cells and debris and add 2 ml of pre-warmed fresh hippocampal medium.

 

**Maintenance**

*Neurobasal media:*

- 500 ml neurobasal (Invitrogen, 12348-017)
- 10 ml B27 serum free supplement (Invitrogen, 17504-044)
- 5 ml Penicillin-Streptomycin  (Invitrogen, 15140-122)
- 1.25 ml L-glutamine (Invitrogen, 25030-149)

Feed the astrocyte-neuron cultures twice a week with neurobasal medium, starting four days after plating. Preincubate the media about 30 min in the incubator in a ventilated flask to equilibrate the temperature and CO_2_.

### Part 2: CALCIUM IMAGING

Hippocampal recording buffer: 110 mM NaCl, 5.4 mM KCl, 1.8 mM CaCl_2_, 0.8 mM MgCl_2_, 10 mM D-glucose, 10 mM HEPES (All chemicals from Sigma) pH 7.4 (adjusted with NaOH).

**Loading calcium indicator dye into astrocytes**

1. Place the culture in a well on a 6-well plate filled with 2 ml hippocampal recording buffer containing 2.5 μM Fluo-4, AM (Invitrogen, F-14217) and 0.05% Pluronic® F-127 20% solution in DMSO (Invitrogen, P-3000MP) and incubate at room temperature for 10-30 min.
2. Remove Fluo-4 solution and wash coverslips 3 times with hippocampal recording buffer and incubate at room temperature for 30 min.
3. Place the coverslip on the chamber, and clean the other side of coverslip with lens cleaning liquid.
4. Put one small drop of immersion oil (Immersion Oil TYPE DF from Cargille) on the objective and put chamber (Open chamber for 25 mm round coverslip from Warner Instruments) on the stage of the microscope (Olympus IX71).
5. Look at the cells using transmission light to see how the cells look, and get them into focus. Then illuminate the cells with 488 nm from a monochromator (Polychrome V from TILL Vision) and determine if the fluorescence signal of Fluo-4 is uniform and detectable in astrocytes.
6. Use EPI and TIRF illumination to measure calcium in astrocytes.

 

**TIRF microscopy**

Briefly, we use an Olympus IX71 microscope equipped with an Andor IXON DV887DCS EMCCD camera.  The control of excitation and image acquisition is achieved using TILLVision software.  The beams of 454/488/515 nm Argon (100 mW) and 442 nm solid state (45 mW) lasers are combined and controlled with a TILL Polyline laser combiner, TIRF dual port condenser and acoustoptical tuneable filter and controller (AOTF; all from TILL Photonics) and fed into a Kineflex broad band fiber for entry into the TIRF condenser. We use an Olympus 60X 1.45 NA lens to achieve TIRF. The camera gain is adjusted for each astrocyte to provide the best signal to noise images. The background and principles of TIRF microscopy have been recently reviewed^4, 5^. Most of the optical components we use were purchased from TILL Photonics, which is now part of Agilent Technologies (http://www.till-photonics.com/). The TIR penetration depth can be calculated from the equations below.


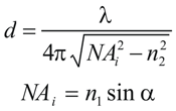


 

d = penetration depth

n1 = refractive index of glass

n2 = refractive index of cell

a = angle of incidence

NAi = numerical aperture of incidence

In order to ensure the laser is aligned optimally for TIRF we find it useful to observe 100 nm fluorescent bead (Invitrogen, F8803). We present still frames and videos of beads with EPI and TIRF microscopy. When in TIRF, one observes a dramatic increase in signal-to-noise and the beads display Brownian diffusion. We find it useful to observe the behavior of 100 nm beads with TIRF microscopy on a regular basis (~once per week) to be sure that optimal TIRF occurs, rather than the compromised oblique illumination that would occur if the critical angle were not equal to α (see Fig 1).

**Application of G-protein coupled receptor agonists**

Astrocytes express a variety of Gq-coupled receptors^6, 7^ including metabotropic glutamate receptors and P2Y receptors (agonist, ATP, ADP). Activation of these receptors leads to significant increases in intracellular calcium levels within astrocytes. For instance, one can readily observe intracellular calcium elevations during application of ATP (30 μM) to astrocytes^8-10^. We use a fast solution switcher from Warner Instruments called the VC-77SP Fast-Step Perfusion System (http://www.warneronline.com/index.cfm). With this method solutions can be applied in less than ~10 ms.


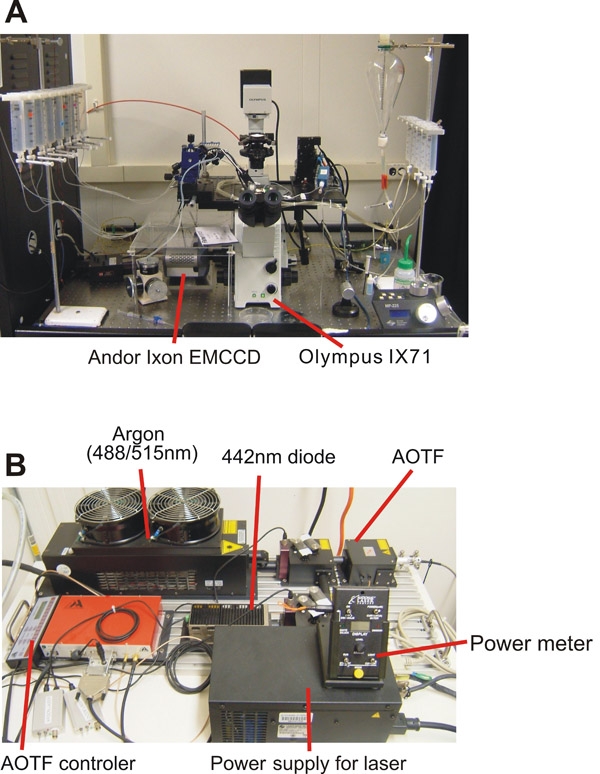
 
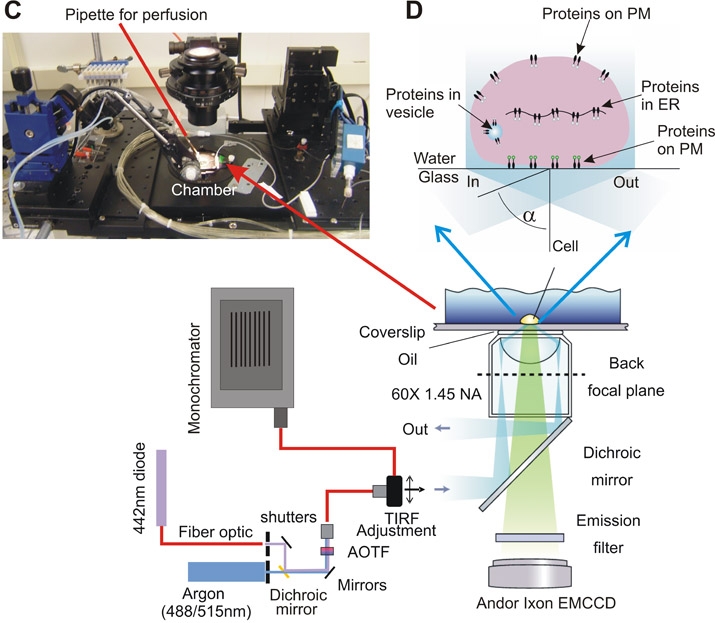


**FIGURE 1.** Cartoon and photographs of the imaging set up. A. Shows a photograph of the microscope mounted on an airtable, whereas (B) shows a photograph of the laser assembly, controllers and beam boxes. C. Shows a photograph of the microscope stage with the chamber mounted for imaging.  On the left the fast perfusion device can be seen (along with the stepper motor and theta tubing). On the right the headstage of an Axopatch 200A amplifier is seen.  The cartoon schematizes the light path in the set up and how TIRF is achieved. The laser is focused on the back focal plane of the 60X 1.45 NA objective lens and its position is adjusted off center so that it emerges into the immersion oil at the critical angle α. At this point the beam suffers from total internal reflection and decays with a distance λ (see equation in main text) into the medium of lower refractive index. In this case this is the recording buffer surrounding the astrocytes and the astrocytes themselves. The result is optical excitation (and thus imaging) of molecules within ~100 nm of the plasma membrane. In the cartoon of the cell this is shown as green “excited” membrane receptors, whereas those within the cell or on the top surface of the cell are not excited. A full account of TIRF microscopy has been provided by Steyer and Almers^4^.


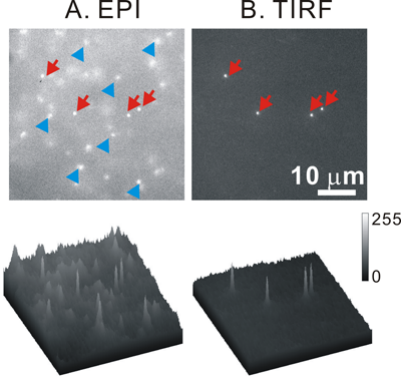


**FIGURE 2.** Images of 100 nm fluorescent beads acquired with EPI and TIRF microscopy. A. Shows EPI images of a field of view with several dozen 100 nm fluorescent beads. The red arrows point to beads that have settled onto the glass coverslip, whereas the blue arrowheads point to beads that are diffusing in water. B. Shows a TIRF image of the same field of view as shown in A. In this view only the adherent beads shown by red arrows are visible. This is because these had settled onto the glass coverlsip and were thus within the ~100 nm evanescent field. The beads shown in A by blue arrows are not within this region and are thus invisible in the TIRF images. The lower plots show 3D rendering of the images. It is clear that a large increase in signal-to-noise occurs for beads within the evanescent field when observed by TIRF microscopy. In fact for these images the signal-to-noise for EPI was 7.1 ± 0.6, whereas for TIRF it was 20 ± 0.7.


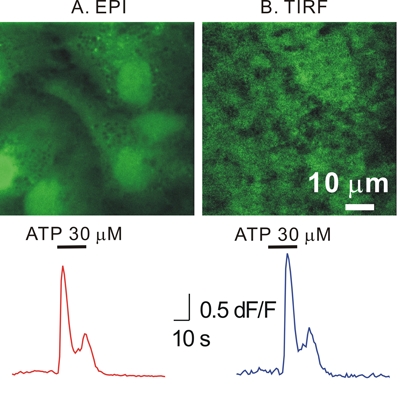


**FIGURE 3.** Images of astrocytes loaded with Fluo-4 calcium indicator dye acquired with EPI and TIRF microscopy.  A. EPI images of a field of view with five astrocytes. B. A TIRF image of the same field of view shown in B. Note that the images in A and B are significantly different. This is because with TIRF illumination only the plasma membrane regions in close apposition to the glass coverslip are imaged.  The lower panels show ATP-evoked intracellular calcium transients imaged with EPI and TIRF microscopy.

## Discussion

It is well-established that astrocytes display intracellular calcium elevations. These occur spontaneously, can be triggered by neuronal activity or by application of agonists to activate receptors on the astrocyte surface^11^. One important and controversial issue is whether astrocyte intracellular calcium elevations can trigger the release of signaling molecules that activate receptors on neurons^11, 12^. This is controversial because there has been evidence for and against this view, as highlighted in the reviews by the Haydon^7, 13^ and McCarthy^11^ labs. Based on our recent brain slice imaging and electrophysiology data we argued that a better and precise understanding of astrocyte calcium dynamics is needed before new hypothesis driven experiments can be designed to determine how astrocytes impact neurons^14^. In this video article we present a simple method to image near plasma membrane and global intracellular calcium changes almost simultaneously in cultured astrocytes. An unavoidable technical requirement of TIRF microscopy is that cultured cells have to be used because they adhere to a glass coverslip within the evanescent field depth^3^. It is worth noting that astrocytes in culture change their gene expression profiles when compared to those in vivo^15^, and so this caveat should be considered when implementing this method. With this consideration in mind however the simple method we report does allow one to image and quantify near plasma membrane and global intracellular calcium changes almost simultaneously. The further application of the approach to astrocytes holds the promise of providing accurate data on intracellular calcium changes near the plasma membrane of astrocytes. The availability of such quantitative data will be useful for the complete understanding of astrocyte biology.
